# Special Issue Introduction: Human Microbiota—Current Updates on Pathogenetic Mechanisms and Methodological Advances

**DOI:** 10.3390/genes15121552

**Published:** 2024-11-29

**Authors:** Valeria D’Argenio

**Affiliations:** 1 Department of Human Sciences and Quality of Life Promotion, San Raffaele Open University, Via di Val Cannuta 247, 00166 Roma, Italy; dargenio@ceinge.unina.it; 2 CEINGE-Biotecnologie Avanzate Franco Salvatore, Via G. Salvatore 486, 80145 Napoli, Italy

Advances in sequencing technologies have made it possible to study microbial communities at a previously unimaginable resolution [1,2,3]. In particular, the human microbiota, defined as the community of microorganisms living inside and on the surface of the human body, has attracted scientists’ attention as a potential player involved in both human health and diseases [1,2,3]. However, despite the huge number of studies published in recent years and the progress made, several questions remain unresolved regarding the composition and functions of the human microbiota.

Indeed, these dynamic microbial communities co-evolved together with their human host, establishing a mutualistic relationship where microbes perform crucial functions, such as in metabolic reactions, immune system development, pathogen defense, and behavioral responses [4].

Due to the existence of this intricate network of crucial interactions between humans and their microbiota, it is easy to imagine that each factor impairing this relationship, thus affecting microbiota composition and/or abundance, may impair human physiology. In line with this hypothesis, microbial dysbiosis has been observed in an incredibly wide range of illnesses. This explains the exponential increase in metagenomic research observed in the last decade.

An additional intriguing aspect of human microbiota research is that it not only does it allow us to better elucidate the molecular mechanisms underlying human diseases development and progression, it also represents a modifiable factor that may be easily modified by targeted therapies [1,2,3,4]. However, an important factor limiting the further development of this field is the exact definition of the so-called “healthy microbiota”. Indeed, it is now well known that the human microbiota colonizes almost all our body and that microbial genes, i.e., the human microbiome, are more abundant than those present in our genome and represent its functional expansion; indeed, microbial genes are able to carry out functions that would not be provided otherwise [4]. It has to be emphasized that different microbial communities are established at different sites in the body, based on different environmental factors and contributing to different functions. Moreover, a high intra- and inter-individual variability has been reported in human microbiota composition. In this context, the concept of “core microbiota”, understood as a group of microbial taxa constantly present at a specific body site, has been coined as the opposite of the “transient microbiota”, which, being influenced by different stimuli (such as diet, physical activity, stress, and traveling), varies over time [4]. A recent report by Van Hul and colleagues tried to define the healthy gut microbiome, since most of the human microbiota live in the gut and this community has been recognized as the most crucial to human health [5]. The first issue that they raised was the question regarding the definition of the healthiest population, and then they tried to define the healthy gut, considering the complex functions that it plays, beyond the microbiota. As expected, it emerged that a universally accepted definition of a healthy gut microbiome is really difficult to achieve, considering the number of factors affecting its high variability. Indeed, even if some indicators of healthy status have been identified, a consensus has still not been reached on what constitutes a healthy gut microbiota [5].

Moreover, it should also be highlighted that we need to move from observational studies to mechanistic ones in order to identify causation instead of correlation.

This means that despite the huge number of data produced, several challenges remain, and future research, including longitudinal studies and multidisciplinary and multi-omics approaches, are required to address these issues.

In this context, this Special Issue of the journal Genes was launched to explore all these aspects, focusing not only on the identification of specific microbial imbalances in association with a disease but also on the molecular mechanisms underlying this relationship and on the technical challenges and latest advances in metagenomic studies.

With regard to the latter aspect, Terrón-Camero and colleagues, inspired by the increasing interest in metagenomic studies, summarized the currently available methodological strategies for microbiome analysis, also including bioinformatic tools [6]. In particular, they highlighted both the sequencing of hypervariable regions and shotgun sequencing as strategies that enable the taxonomic classification of the microorganisms present in a microbial environment, but they were not able to evaluate whether these microorganisms were actively expressed. Thus, they suggested that meta-transcriptomics, which overcomes this limitation and also allows the study of host–microbe interactions, should be the preferred approach in this field. Finally, they also developed two pipelines for metagenomics and meta-transcriptomics data analysis and discussed their performance after testing them on both simulated and experimental datasets [6].

Other papers included in this Special Issue have focused on the relationship between the microbiota and the presence of a specific disease. In particular, the role of the gut microbiota has been widely explored in association with different conditions.

In this context, D’Argenio et al. focused on the role of the gut microbiota and its association with neurodegenerative disorders [7]. As mentioned above, the gut microbiota has been claimed to be a key factor in human health, and its alteration has been reported in several diseases, including Alzheimer’s disease (AD). In this study, the authors aimed to analyze both the gut microbiome and mycobiome in an AD mouse model, the 3xTg-AD mouse, to identify AD-specific microbial alterations. Interestingly, in addition to significant differences in bacterial composition between the AD and wild-type mice, they also found a significant decrease in the fungal *Dipodascaceae* family, suggesting the importance of metagenomics going beyond the bacterial counterpart [7]. This study not only has the overall advantage of having identified significant differences in the gut microbiota composition between AD and wild-type mice but also represents a starting point for further studies aiming to assess whether AD treatments act by changing the gut microbiota or, conversely, whether gut microbiota manipulation may have beneficial effects in AD.

Continuing on the topic of neurodegenerative diseases, the review by Kiriyama et al. focuses on bile acid modifications induced by the gut microbiota and their role in neurodegenerative disease development [8]. Indeed, various microorganisms can modify bile acids in the gut, meaning that a variety of bile acids exist in our body, exerting different functions and being able to recognize different cellular receptors. In particular, some bile acids have been reported to be able to affect mitochondrial functions and induce autophagy progression; both of these dysfunctions are present in several neurodegenerative diseases, such as AD, Parkinson’s disease, and Huntington’s disease. Thus, bile acid production modulation may be a mechanism through which the dysbiotic gut microbiota present in these patients may affect neurons [8]. Of course, further studies are required to assess this hypothesis. If confirmed, this could open the door to the development of novel therapeutic strategies for the management of neurodegenerative disorders.

Alsharairi and colleagues explore the relationship between early-life gut microbiota dysbiosis and susceptibility to neonatal diabetes [9]. In particular, they review the mechanisms linking diet to gestational diabetes mellitus, epigenetic alterations (such as DNA methylation), and gut microbiota dysbiosis. Indeed, DNA methylation in the cord blood of newborns exposed to gestational diabetes is altered, and this influence gene expression, including genes involved in glucose metabolism. The authors identified seven genes whose expression was significantly altered in neonatal diabetes as a consequence of gestational diabetes exposure, with four being upregulated (*TRIB1*, *POU2F1*, *PON1*, and *TXNIP*) and three being downregulated (*PGC1α*, *MEST*, and *NRF2*). Gut microbiota alterations in neonatal diabetes are still poorly investigated; however, maternal diet and gestational diabetes have been associated with gut microbiota dysbiosis in newborns. This may be mediated by epigenetic mechanisms that are not yet completely understood [9]. Interestingly, this mechanism could also be translated to other disorders, opening the door to a number of possible studies linking epigenetics to microbiota colonization and composition.

The bi-directional interaction between the host genome and those of the microbes is the object of an interesting review by Boccuto and colleagues [10]. Intriguingly, they highlight that artificial intelligence algorithms, allowing for big data analysis, were able to identify human genes encoding enzymes, inflammatory cytokines, and proteins that had similarity with those of the gut microbiome. Moreover, epigenetic and non-epigenetic mechanisms mediated by gut microbes and affecting the expression of human genes are also reviewed. Indeed, the possibility of reprogramming the metagenome through pre-, pro-, and postbiotics, fecal microbiota transplantation, and antibiotic use implies effects on both of these types of mechanism, so understanding them is crucial in moving towards even more personalized medicine [10].

Even though the gut microbiota plays a key role in human health acquisition and maintenance, microbes live in almost every part of our body, and the alteration of other microbial niches can also contribute to the development of specific disorders.

In particular, Veneruso et al. explored the possible association between semen microbial alterations and infertility [11]. The authors were able to highlight significant differences between the semen microbiome of healthy and infertile men and also found an intriguing correlation with semen hyperviscosity, suggesting a mechanism through which microbes may affect fertility [11].

Finally, the therapeutic potential of microbial manipulation has also been explored. In particular, Peretti and colleagues reviewed the current evidence on pharmacomicrobiomics applied to the treatment of rheumatoid arthritis and spondylarthritis [12]. They also found, in this case, an intriguing mutual and reciprocal influence between the microbiota and drugs that, once it is better understood, may ameliorate rheumatic diseases management, supporting personalized treatments to optimize efficacy and limit side effects and/or drug failure.

In conclusion, the heterogeneity of the papers included in this Special Issue reflects the wide range of aspects linking the human microbiota to its human host, which have led to great interest from the scientific community in exploring further. Several aspects have been elucidated to date, but many others require further study. The development of novel technologies, including new analytic strategies and bioinformatic tools, will aid further research in this area.
